# Lignans intake and enterolactone concentration and prognosis of breast cancer: a systematic review and meta-analysis

**DOI:** 10.7150/jca.55477

**Published:** 2021-03-10

**Authors:** Zhen Liu, Yin-Jiao Fei, Xin-Hui Cao, Di Xu, Wen-Juan Tang, Kai Yang, Wen-Xiu Xu, Jin-Hai Tang

**Affiliations:** 1 Department of General Surgery, the First Affiliated Hospital of Nanjing Medical University, Nanjing 210029, P.R. China.; 2 School of Clinical Medicine, Xuzhou Medical University, Xuzhou 221000, P.R. China.

**Keywords:** lignans, enterolactone, breast cancer, Meta-analysis.

## Abstract

**Background:** Some literature has studied the relationship between lignans intake and its metabolite, enterolactone, and breast cancer survival, but the results are far from consistent and conclusive. Therefore, we conducted a systematic review and meta-analysis in this situation.

**Methods:** From its inception to August 2020, we conducted a comprehensive search of PubMed, Embase, Web of Science, and Cochrane Library databases. This study reported the correlation between lignans intake and serum enterolactone concentrations and prognosis of breast carcinoma. The total hazard ratios (HRs) and 95% confidence interval (95% CI) were estimated, comparing the highest versus the lowest category of lignans intake and serum enterolactone concentrations, using a fixed or random-effects effect model.

**Results:** A total of 6 articles were included in reporting the all-cause mortality (ACM), breast cancer-specific mortality (BCSM), and recurrence of 2668, 1516, and 474 breast cancer patients in 18053 breast cancer patients. In postmenopausal women with breast cancer, lignans intake or enterolactone concentrations were associated with a reduced risk of all-cause mortality (maximum and minimum) (pooled HR = 0.73, 95% CI, 0.58-0.91), as was the association with breast cancer-specific mortality (maximum and minimum) (pooled HR = 0.72, 95% CI, 0.60, 0.87). Stratified analysis showed that exposure type and diagnosis time might be the sources of heterogeneity. In premenopausal women, the relationship seemed to be the opposite, showing an increased risk of all-cause mortality (maximum and minimum) in breast cancer patients (pooled HR = 1.57, 95% CI, 1.11-2.23). No significant association was found between lignans intake or enterolactone concentrations and breast cancer recurrence (pooled HR = 0.91, 95% CI, 0.69, 1.20).

**Conclusion:** This study provides limited evidence that lignans intake and higher serum enterolactone concentrations in postmenopausal women are beneficial to breast cancer patients' prognosis. In premenopausal women, however, the relationship may be reversed.

## 1. Introduction

In 2020, the American Cancer Society estimated that the number of new breast cancer cases and deaths that will occur in the United States is equivalent to the data estimated in 2019 1, 2; for women, breast cancer alone accounted for 30% of female cancers 2, also the leading cause of cancer death 3. Therefore, it is a significant public health issue to find factors that increase survival and reduce recurrence in breast cancer patients.

Diet and nutrition are recognized modifiable risks of breast cancer 4; its relationship with breast cancer patients has attracted people's attention. Among them, the relationship between phytoestrogens and breast cancer patients has also attracted people's interest 5, 6. Phytoestrogen is a non-steroidal plant-derived xenoestrogen that mimics circulating estrogen in structure or function 7. For example, a study reported that the intake of phytoestrogens soy and isoflavones before diagnosis was associated with a small reduction in postmenopausal breast cancer overall survival 8. Lignans are also important phytoestrogens in the Western diet. It is metabolized in the body into biologically active forms, enterolactone and enterodiol 9. Considering the reduced survival rate caused by breast cancer, many studies have explored the relationship between lignans and its metabolites and breast cancer 10, 11. However, due to individual studies' limitations, the results of individual studies were inconsistent or contradictory. In Jaskulski's study, lignans intake has a protective effect on the prognosis of postmenopausal breast cancer 11, While Fink's investigation was not consistent with the conclusion of this research 12. To clarify these contradictory or inconsistent results and to more accurately assess the relationship between breast cancer and lignans and its metabolites, we conducted a meta-analysis of published studies. It is generally believed that meta-analysis is a powerful statistical tool to overcome the limitations of different sample sizes in individual studies and produce the best estimates. The previous systematic review dates back to 2014, and several documents have been published after this date. The purpose of this work is to systematically review and meta-analyze the effects of lignans and its metabolites on the prognosis of breast cancer patients.

## 2. Methods

### 2.1. Search Strategy

We searched for articles published before August 2020 in PubMed, Embase, Web of Science, and Cochrane library databases. We identified studies by using Medical Subject Headings (MeSH), the keywords used for the systematic search were: (“breast carcinoma” OR “breast cancer”) AND (mortality OR survival OR death OR prognosis OR relapse) AND (enterolactone OR lignan), and the specific search formula was given in Supplementary. We would also search by combining the above terms with keywords and reference manuals. This investigation was registered on* PROSPERO* under ID number CRD42020208054 (https://www.crd.york.ac.uk/prospero/).

### 2.2. Selection criteria

We used the following criteria to include eligible articles in this review: (1) research conducted in women diagnosed with breast cancer, (2) report the intake of lignan or the serum level of enterolactone (either before or after diagnosis) as exposure, in at least two categories, (3) report all causes or mortality specific to breast cancer as a result of interest, (4) provide an estimate of the effect as a relative risk (RR) or hazard ratio and the 95% confidence interval (CI) of the corresponding mortality rate for each category, and(5) studies on lignan intake need to have a relatively strict dietary assessment, such as FFQ(food frequency questionnaire), or use a country or center-specific nutritional questionnaire. Besides, articles were excluded if (1) they were abstracts, letters, reviews, or Nonclinical studies, (2) papers with insufficient data for estimating hazard ratio (HR) and 95% confidence interval (Cl), (3) research had duplicate data or repeat analysis.

### 2.3. Data Extraction

All qualified documents were evaluated and extracted by two independent researchers (LZ and FYJ). If there were conflicting opinions, the two researchers would discuss, seek a third person's views (CXH), and reach a consensus. The included studies included the following characteristics: study name, first author's name, publication date, country, number of subjects/cases, exposure name and level classification, average follow-up time, menopausal status, hazard ratios(HR) of all-cause mortality (ACM), breast cancer-specific mortality (BCSM) and recurrence and 95% confidence intervals (CI), as well as the variables, used to maximize the adjustment of the multivariate analysis. Finally, we chose ASM, BCSM, and recurrence as our endpoints for meta-analysis (Table 1). The factors adjusted for HR and 95% CI calculated by each study are summarized in Supplementary.

### 2.4. Quality Assessment

**Figure 1:**
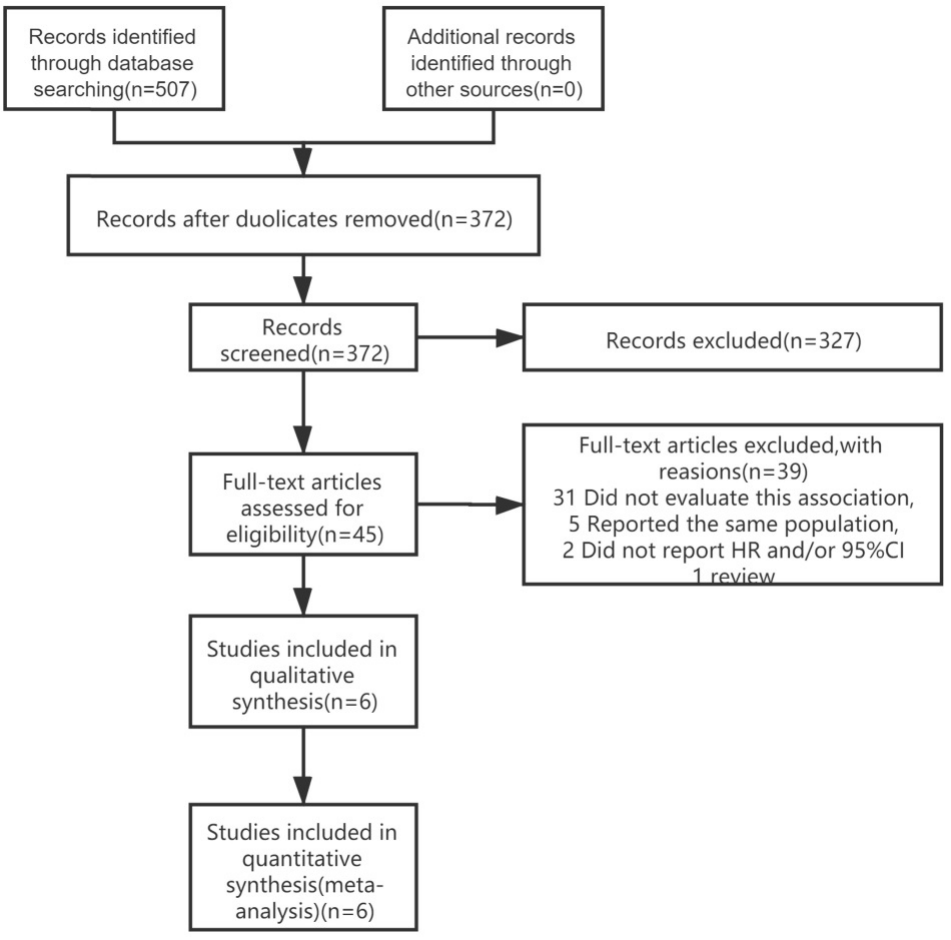
Literature search and study selection process for inclusion in the meta-analysis of lignans intake or enterolactone in vivo and outcomes of breast cancer.

The Newcastle-Ottawa Scale (NOS) was used to assess the included studies' quality by two independent authors (LZ and FYJ). The NOS score consists of three parts, namely selection (0-4 points), compatibility (0-2 points), and outcome assessment (0-3 points) (Supplementary). Research with NOS score ≥ 6 points was considered a high-quality study and included in the analysis, and finally, six articles were included in the study (Figure 1).

### 2.5. Statistical analysis

As an index to measure the value of prognosis, HR>1 means that there is a poor prognosis for the maximum group of lignans intake or serum enterolactone levels. Between included trials' heterogeneity was assessed using Cochrane's Q test and quantified by I^2^ statistic, which describes the percentage of the total variation in studies due to heterogeneity rather than chance (A P heterogeneity <0.10 or I^2^ >50% suggested significant heterogeneity). When the heterogeneity is significant, the random-effects model is used; otherwise, the fixed effects model can combine the effect sizes. To test each main study's impact on the combined effect's size, we performed a sensitivity analysis by continuously excluding each study from the primary analysis. Also, we did a subgroup analysis to explore the source of heterogeneity. Although the number of initial studies was small (<10), we still perfected the egger test to investigate potential publication bias. All analyses were conducted with Stata software, version 12 (Stata Corp, College Station, USA). A P value <0.05 was considered statistically significant.

## 3. Results

### 3.1. Study selection

We identified 507 publications in 4 databases (PubMed, EMBASE, Web of Science, Cochrane library). After reviewing the titles and abstracts of the retrieved publications and removing duplicates, 45 publications were retained for full-text screening. After excluding studies that did not meet the inclusion criteria, a total of 6 articles 12-17 were included in the meta-analysis (Figure 1). The most common reasons for exclusion were lack of data on lignans intake or enterolactone in vivo with BC prognosis (n = 31 studies). Five studies reported the same clinical trials and populations as the literature included in the meta-analysis, so they were excluded 10, 11, 18-20.

### 3.2. Characteristics of studies

Table 1 summarized the characteristics of the six articles (three cohort studies, three case-control studies). In general, all papers had all-cause mortality (ACM) and breast cancer-specific mortality (BCSS) data, including two articles on recurrence 15, 17. The reports were published between 2007 and 2017. These studies were conducted in the following geographic regions: the United States, 2; Europe, 4. Of the six studies, four reported the relationship between lignans intake or the enterolactone level in vivo before and after menopause and breast cancer outcomes. The remaining 2 reported the relationship between serum enterolactone levels after menopause and breast cancer outcomes 15, 17.

#### 3.2.1. Premenopausal lignans intake or enterolactone in vivo and risk of all‑cause and breast cancer-specific mortality

**Figure 2:**
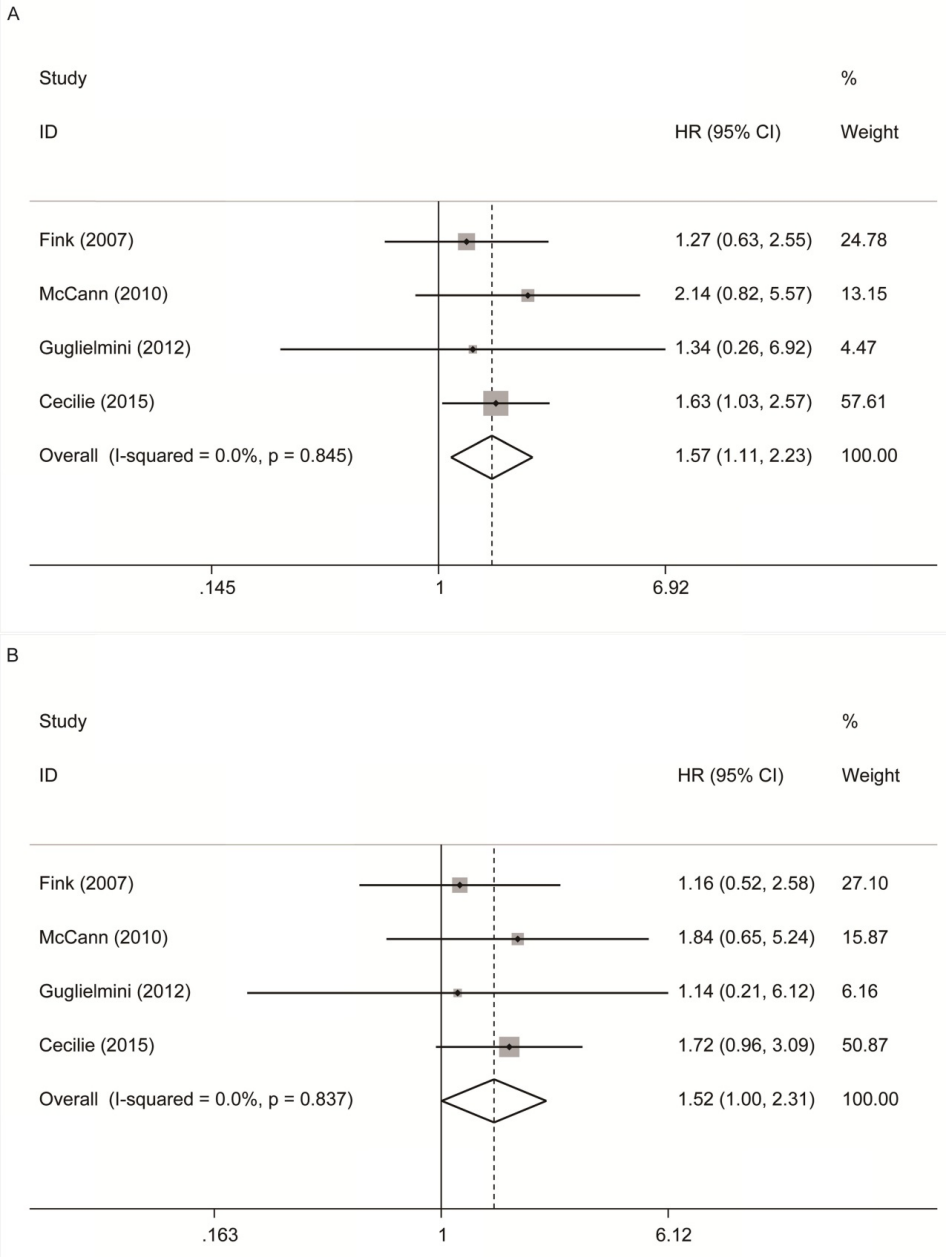
Premenopausal lignans intake or enterolactone in vivo and risk of all‑cause and breast cancer-specific mortality. (A) risk of all‑cause mortality, (B) risk of breast cancer-specific mortality.

As shown in Figure 2A, 4 studies 12-14, 16 reported the relationship between premenopausal lignans intake or enterolactone in vivo and all-cause mortality, and there was a significantly increased risk association (maximum vs. minimum) , and no significant heterogeneity was found (summary HR = 1.57, 95% CI, 1.11-2.23; P_heterogeneity_= 0.845, I^2^ = 0.00%); These 4 studies also reported the relationship between premenopausal lignans intake or enterolactone in vivo and breast cancer-specific mortality(Figure 2B), and there was no significant correlation (maximum vs. minimum) (summary HR = 1.52, 95% CI, 1.00-2.31; P_heterogeneity_= 0.837, I^2^ = 0.00%). Because there was no large heterogeneity, the fixed-effects model was used to combine study-specific results.

#### 3.2.2. Postmenopausal lignans intake or enterolactone in vivo and risk of all‑cause and breast cancer-specific mortality

**Figure 3:**
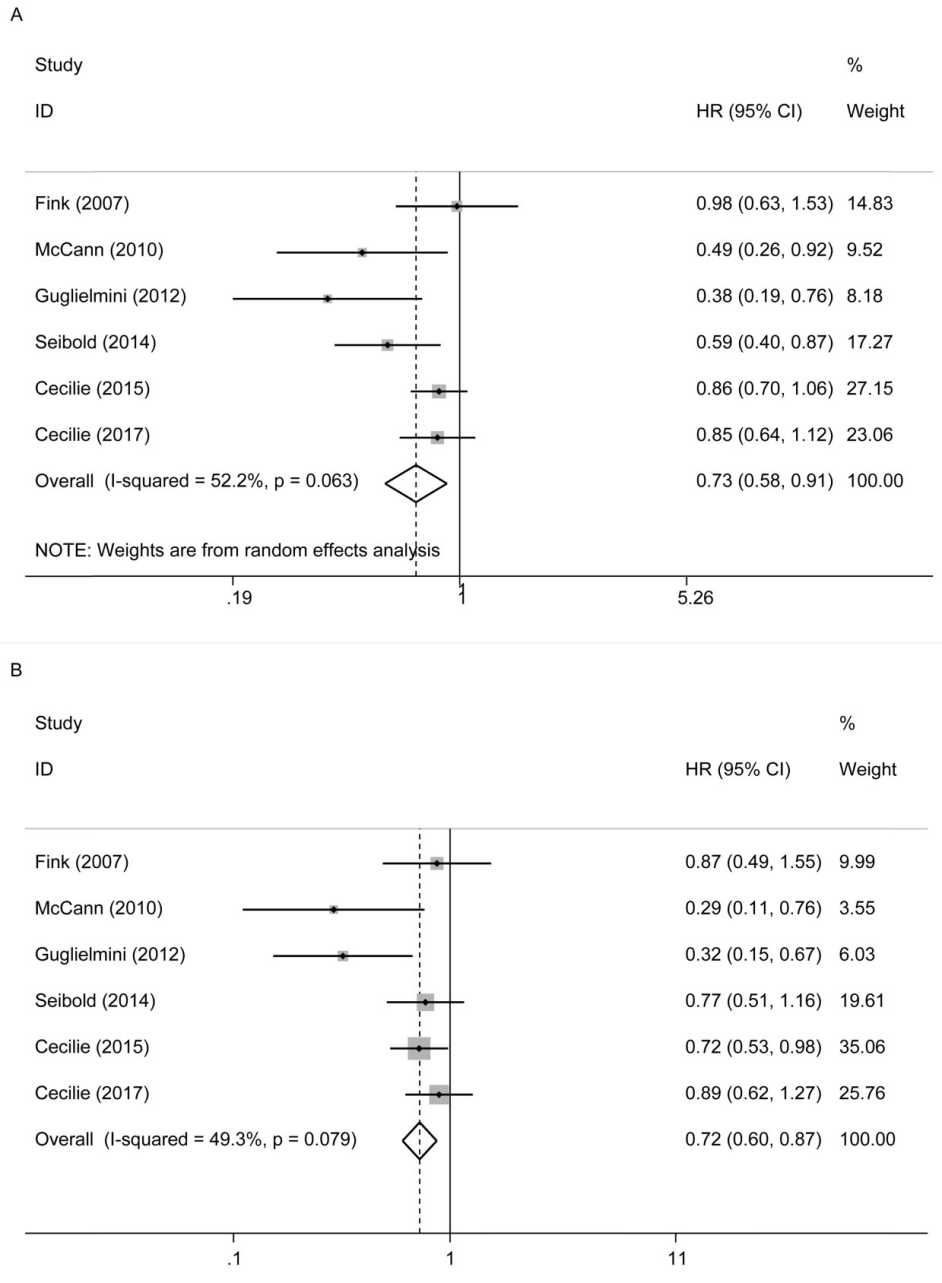
Postmenopausal lignans intake or enterolactone in vivo and risk of all‑cause and breast cancer-specific mortality. (A) risk of all‑cause mortality, (B) risk of breast cancer-specific mortality.

**Figure 4:**
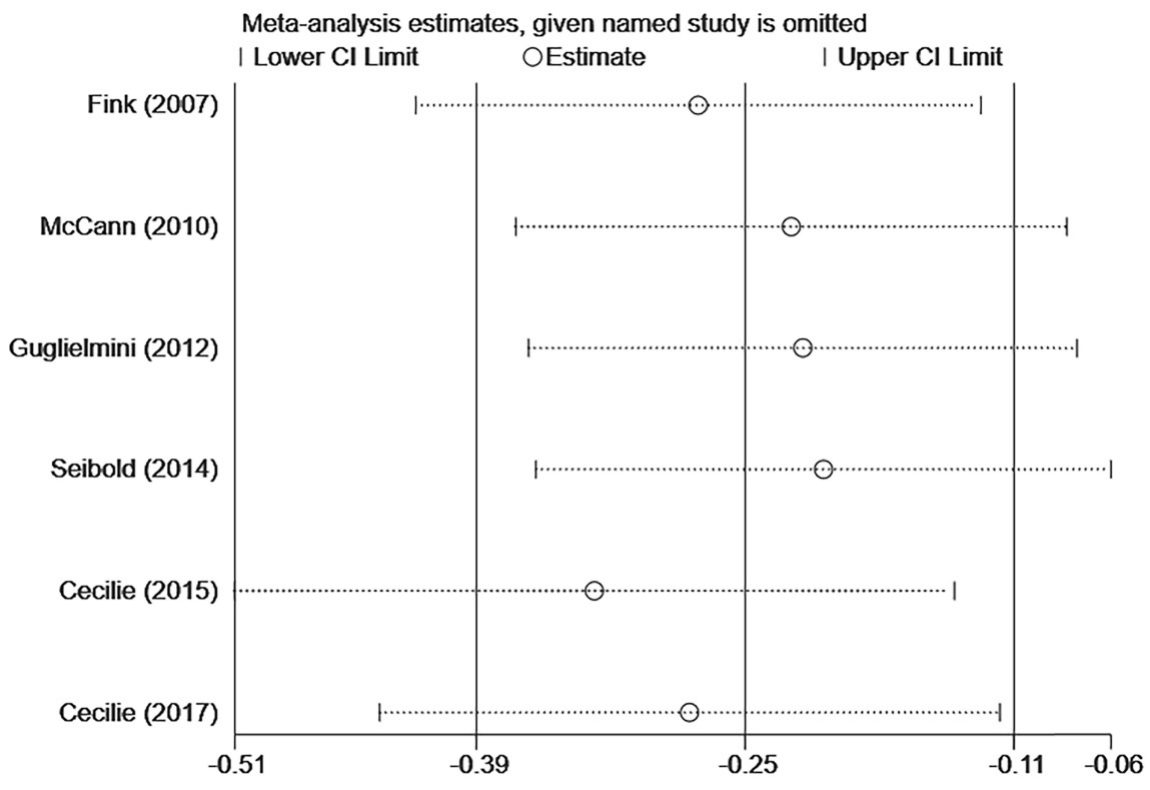
Sensitivity analysis of postmenopausal lignans intake or serum enterolactone and ACM.

In Figure 3A, six studies 12-17 reported the relationship between lignans intake or enterolactone in vivo and all-cause mortality in postmenopausal breast cancer patients. They had a significant risk reduction relationship (maximum vs. minimum) (summary HR = 0.73, 95% CI, 0.58-0.91), and the heterogeneity was also significant (P_heterogeneity_= 0.063, I^2^ = 52.2%). Because of the large heterogeneity, we adopted a random-effects model to combine study-specific results. We used subgroup analysis to trace potential sources of heterogeneity between studies and assess the consistency of conclusions between different patient subgroups. Table 2 presented the results of subgroup analyses. We conducted subgroup analysis from three aspects, the type of study, the type of exposure, and the time of diagnosis. The heterogeneity of the subgroups of different research types was still very large (I^2^ of cohort:60.0%, Ph=0.082; I^2^ of case-control: 51.2%, Ph=0.127), the heterogeneity of exposure types is reduced (I^2^ of lignans intake:40.3%, Ph=0.187; I^2^ of serum enterolactone: 64.4%, Ph=0.060), and the heterogeneity of different diagnosis times is significantly reduced (I^2^ of pre-diagnosis:10.6%, Ph=0.34; I^2^ of post-diagnosis: 15.1%, Ph=0.278). It can be inferred from the above that the source of heterogeneity has little to do with the type of research, and the type of exposure and the time of diagnosis may be the source of heterogeneity. We also evaluated the stability of the results through sensitivity analysis (Figure 4), and the results showed that the overall effect is stable.

In Figure 3B, these six studies also reported their relationship with breast cancer-specific mortality. There was also a reduced risk relationship between them (maximum vs. minimum) (summary HR = 0.72, 95% CI, 0.60, 0.87). We used a fixed-effects model to combine the research results, for there was no significant heterogeneity in the research (P_heterogeneity_= 0.079, I^2^ = 49.3%).

P_d_: P values represent differences between subgroups; Ph: P values for between-study heterogeneity.

#### 3.2.3. Enterolactone in vivo and breast tumor recurrence

Two papers 15, 17 studied the relationship between postmenopausal enterolactone in vivo and breast tumor recurrence (Supplementary). To summarize the two articles by Seibold et al. and Cecilie et al., postmenopausal lignans intake or serum enterolactone were not associated with the risk of breast tumor recurrence (maximum vs. minimum: summary HR = 0.91, 95% CI, 0.69-1.20, P_heterogeneity_ = 0.272, I2 = 17.2%). Seibold's study was conducted in patients after diagnosis, while Cecilie's research was conducted in pre-diagnosed populations. They concluded that the level of enterolactone in the body has nothing to do with breast cancer recurrence.

### 3.3. Publication bias of studies

**Figure 5:**
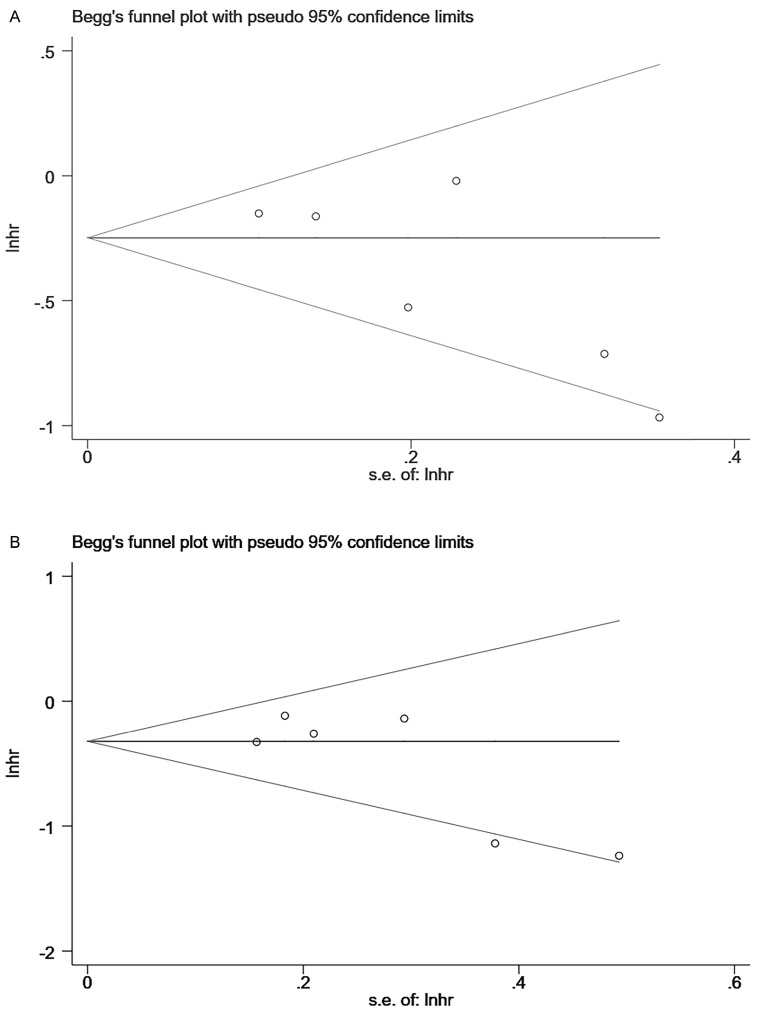
Begg's tests of association between postmenopausal lignans intake or serum enterolactone and ACM (A) and BCSM (B).

Begg and Egger's tests indicated no publication bias in the meta-analyses for the association between lignans intake or serum enterolactone and all-cause mortality and breast cancer-specific mortality. Although there was little literature included in the review (n=6<10), we still performed these two tests to detect publication bias. Among them, publication bias was not detected for the association between premenopausal lignans intake or serum enterolactone and all-cause mortality (Pr >|z|=1 for Begg's tests and P >|t|=0.975 for Egger's tests); so was breast cancer-specific mortality (Pr >|z|=0.734 for Begg's tests and P >|t|=0.611 for Egger's tests). Publication bias was also not detected for the association between postmenopausal lignans intake or serum enterolactone and all-cause mortality (Pr >|z|=0.133 for Begg's tests and P >|t|=0.087 for Egger's tests), and breast cancer-specific mortality (Pr >|z|=0.452 for Begg's tests and P >|t|=0.093 for Egger's tests). Figure 5 was the Begg's tests of association between postmenopausal lignans intake or serum enterolactone and all-cause mortality (Fig. 5A) and breast cancer-specific mortality (Fig. 5B).

## Discussion

Many studies have investigated the relationship between lignans intake or the concentration of enterolactone in its blood metabolites and the prognosis of breast cancer. Still, the conclusions were not entirely consistent and accurate. Therefore, we reviewed the published literature and conducted a meta-analysis to make a more precise estimate of this relationship. Our meta-analysis combined the outcomes of 18053 breast cancer patients from 6 individual studies, indicating that in postmenopausal breast cancer patients, the intake of lignans or the concentration of enterolactone in the body has a reduced risk relationship with the patient's all-cause mortality (maximum vs. minimum) (summary HR = 0.73, 95% CI, 0.58-0.91), as was the relationship with breast cancer-specific mortality (maximum vs. minimum) (summary HR = 0.72, 95% CI, 0.60, 0.87). Subgroup analysis also showed that the type of exposure and time to diagnosis might be the source of heterogeneity, rather than the kind of study design. In premenopausal women, this relationship seemed to be the opposite, showing a trend of increased risk with all-cause mortality in breast cancer patients (maximum vs. minimum) (summary HR = 1.57, 95% CI, 1.11-2.23).

In our research, the aggregated estimates were stable when limited to the included articles and seemed to explain some problems. In postmenopausal women, breast cancer patients tended to have a good prognosis when the intake of lignans or enterolactone concentration in the blood is higher. McCann's study has similar conclusions 13: women's dietary lignans intake after the diagnosis of breast cancer was negatively correlated with mortality; Seibold's research team also reached similar conclusions in 2014, and the meta-analysis they did at that time also can prove that this relationship is negatively correlated in postmenopausal breast cancer patients 15. After several follow-ups, in 2020, Seibold's team discovered that a higher enterolactone after diagnosis might benefit postmenopausal breast cancer patients within four years after diagnosis. After this time, this relationship became no longer significant 10. However, Cecilie's study found no apparent correlation between the plasma enterolactone concentration before diagnosis and the prognosis of breast cancer 17. The difference in conclusions may come from diagnosis and the different types of the exposure recorded. In premenopausal women, the higher the intake of lignans or the higher levels of enterolactone in the body seemed to increase the all-cause mortality of breast cancer patients; at this point, only one study in 2015 was consistent with our research conclusions 16. But for this conclusion, the researchers in this article had many thoughts. Firstly, patients may change their eating habits after diagnosis; secondly, they did not fully consider the data on treatment 16. The large number of people involved in this 2015 study accounted for a large proportion of the fixed-effects model, so more research was needed to prove this conclusion.

In this study, we also explored the relationship between the upper and lower quartile lignans intake or the concentration of enterolactone levels and breast cancer prognosis. We provided a meta-analysis of studies 13, 15-17 with quartile level data (Supplementary). In postmenopausal breast cancer patients, the intake of lignans or the concentration of enterolactone in the body had a reduced risk relationship with the patient's all-cause mortality(upper quartile vs. minimum)(summary HR = 0.80, 95% CI, 0.69-0.91); (lower quartile vs. minimum)(summary HR = 0.77, 95% CI, 0.68-0.88).As was the relationship with breast cancer-specific mortality(upper quartile vs. minimum)(summary HR = 0.75, 95% CI, 0.62-0.90).In premenopausal breast cancer patients, we did not find a correlation between them. This seemed to confirm that the intake of lignans and higher serum enterolactone concentration is beneficial to breast cancer patients' prognosis in postmenopausal women.

Polyphenolic phytochemicals (such as lignans) can block and inhibit carcinogenesis and interfere with cell and molecular processors at different carcinogenesis stages. They may also inhibit the activation of pre-carcinogens into electrophilic species and their subsequent interaction with DNA to prevent the occurrence of cancer 21. For example, it can inhibit breast cancer growth and enhance chemotherapeutics' toxicity to tumors in vitro 22, 23. Lignans can be absorbed and metabolized by intestinal microflora into enterolactone. The intestinal microflora's composition and activity seemed to be the most critical factor affecting the difference in plasma enterolactone concentration from person to person, followed by the use of antibiotics 24. In the future, it may be necessary to combine the intestinal microflora to make a more accurate estimation of the factors affecting the concentration of intestinal lactone. It also played a role in inhibiting the proliferation, migration, and metastasis of breast cancer cells in vitro and lung cancer 25-27. All this showed that, to a certain extent, lignans and its metabolites might harm breast cancer.

This research's strengths included a comprehensive and systematic literature review of relevant literature on lignans and its metabolites and breast cancer prognosis. In the postmenopausal women group, we used subgroup analysis to find the source of heterogeneity and used sensitivity analysis to ensure the results' stability, and there is no publication bias. It is also worth talking about the limitations of our literature. First, there was a lack of uniform standards for assessing lignans intake included in the literature; second, dietary supplements also had a particular impact on phytoestrogens. We did not consider the effect in this regard. Finally, we had no fixed limit on the follow-up time; most of them focused on 4-5 years. Future research may need to pay attention to the length of follow-up time. For Guglielmi's analysis 14, we also selected the data from the first five years to study. The relationship we looked at may change with the extension of follow-up time, which was also mentioned in Jaskulski's 2020 study 10.

## Conclusion

In postmenopausal women, lignans' intake and higher serum enterolactone concentration were beneficial to breast cancer patients' prognosis. However, in premenopausal women, such exposure may not benefit the prognosis of the patient. Since there are not many conforming documents included, the conclusions need the support of multi-center, well-designed studies to understand better the potential benefits or harms of lignans and its metabolites to breast cancer patients.

## Supplementary Material

Supplementary figures and tables.

## Figures and Tables

**Table 1:** Characteristics of studies on the association between lignans intake or enterolactone in vivo and outcomes of breast cancer

Study, Author, Publication Year (Ref)	Type of Study	Years of diagnosis, Country	N_total_ (all deaths/due to BC/ recurrence)	Follow-up Time(median)	Exposure type and time	Menopausal status	Categories	All-cause mortality HR(95%CI)	Breast cancer-specific mortality HR(95%CI)	Recurrence
LIBCSP Fink 2007^12^	case-control study	1996-1997 2002-2004 US	1,210 (173/113)	5 years	Dietary Lignans, Pre-diagnosis	Premenopausal (376/43/34)	Max* (≥9) vs. Min* (≤2.2 mg/d)	1.27(0.63-2.54)	1.16(0.52-2.58)	
						Postmenopausal (834/130/79)	Max (≥9) vs. Min (≤2.2 mg/d)	0.98(0.63-1.54)	0.87(0.49-1.55)	
						Total (1210/173/113)	Max (≥9) vs. Min (≤2.2 mg/d)	1.03(0.71-1.49)	0.95(0.60-1.51)	
WEB McCann 2010^13^	case-control study	1996-2001 US	1,122 (160/94)	87.3 months(mean)	Dietary Lignans, Pre-diagnosis	Premenopausal (315/44)	>257 vs. <128 ug/d	2.14(0.82-5.56)	1.84(0.65-5.27)	
						Postmenopausal (807/116)	>318)vs. <155 ug/d	0.49(0.26-0.91)	0.29(0.11-0.76)	
Guglielmini 2012^14^	retrospective cohort study	1984-1991 Italy,	300 (180/112)	0-5 years	Serum enterolactone, Post-diagnosis	Premenopausal	≥10 vs <10 nmol/L	1.34(0.26-6.93)	1.14(0.21-6.05)	
						Postmenopausal		0.38(0.19-0.76)	0.32(0.15-0.66)	
				5-10 years		Premenopausal		1.85(0.49-6.93)	1.77(0.46-6.86)	
						Postmenopausal		0.48(0.28-0.82)	0.52(0.29-0.94)	
MARIE Seibold 2014^15^	case- control study	Germany, 2001-2005	2,182 (269/194/207)	5.4 years	Plasma enterolactone, Post-diagnosis	Postmenopausal	Max (>45.1) vs. Min (≤8.5 nmol/L)	0.59 (0.40-0.87)	0.59(0.37-0.94)	0.77 (0.51-1.16)
							per 10 nmol/L	0.94 (0.90-0.98)	0.94(0.89-0.99)	0.99 (0.95-1.02)
EPIC Cecilie 2015^16^	Prospective multi-center cohort study	European 2004-2009	11782(1482/753)	4 years	Lignans intake Pre-diagnosis	Premenopausal	>2.0mg/d vs <1.1mg/d	1.63 (1.03,2.57)	1.72(0.96-3.09)	
						Postmenopausal	>2.0mg/d vs <1.1mg/d	0.86(0.70-1.06)	0.72(0.53,0.98)	
Cecilie 2017^17^	Cohort study	Denmark 1993-2014	1457(404/250/267)	8 years	Plasma enterolactone Pre-diagnosis	Postmenopausal	≥36.9 v ≤9.5nmol/L	0.85(0.65, 1.13)	0.89(0.62, 1.27)	1.05 (0.72, 1.51)
							Linear per doubling (log2) in concentration	0.95 (0.89, 1.02)	0.94(0.86, 1.02)	0.98 (0.90, 1.06)

*Max, Min: Lignans intake or enterolactone were classified into different categories in literature, which refer to the maximum exposure group and the minimum exposure group

**Table 2:** Stratified meta-analyses of postmenopausal lignans intake or serum enterolactone and all-cause mortality

	Fixed-effect model			Random-effect model		Heterogeneity	
Analysis	HR (95%CI)	P (z test)	P_d_	HR (95%CI)	P (z test)	I^2^	Ph
Subgroup1: type of study	0.78 (0.68-0.895)	0.000	0.249	0.726 (0.579-0.911)	0.006	52.2%	0.063
Cohort	0.819 (0.697-0.963)	0.016		0.762 (0.564-1.029)	0.076	60.0%	0.082
Case-control	0.683 (0.523-0.890)	0.005		0.675 (0.454-1.002)	0.051	51.2%	0.127
Subgroup2: type of exposure	0.780 (0.680-0.895)	0.000	0.222	0.726 (0.579-0.911)	0.006	52.2%	0.063
lignans intake	0.838 (0.700-1.004)	0.055		0.810 (0.605-1.085)	0.158	40.3%	0.187
Serum enterolactone	0.704 (0.569-0.873)	0.001		0.629 (0.419-0.946)	0.026	64.4%	0.060
Subgroup3: Diagnosis time	0.780 (0.680-0.895)	0.000	0.015	0.726 (0.579-0.911)	0.006	52.2%	0.063
Pre-diagnosis	0.842 (0.724-0.979)	0.025		0.839 (0.710-0.991)	0.038	10.6%	0.34
Post-diagnosis	0.531 (0.378-0.895)	0.000		0.522 (0.355-0.768)	0.001	15.1%	0.278
