# Supplementary material for: Lignans intake and enterolactone concentration and prognosis of breast cancer: a systematic review and meta-analysis
Source: J Cancer. 2021 Mar 10;12(9):2787–96. doi: 10.7150/jca.55477 (PMC8040718; doi:10.7150/jca.55477)

**Table 1 Characteristics of literature that duplicate the data extracted in the review**										
Study, Publication Year(Ref)	Country, Years of diagnosis	N_{total} (all deaths/ due to BC)	Follow-up Time(median)	Exposure	Menopausal status (Nsubgroup/deaths)	Enterolactone categories	All-cause mortality HR(95%CI)	Cancer-specific mortality HR(95%CI)	recurrence	Adjustment factors
Buck 2011a MARIE	Germany (RNK), 2002–2005	1,140 (162/124)	6.1 years	Serum enterolactone, postdiagnosis	postmenop. (all)	Q4 (≥42.3) vs. Q1(≤7.8 nmol/L)	per 10 nmol/L 0.58 (0.34–0.99) 0.94 (0.88–1.00)	-		TNM, grade, ER/PR, mode of detection, diabetes, use of menopausal hormone therapy, BMI, physical activity (strata: age, study region

Buck 2011b MARIE	Germany, 2001–2005	2,653 (321/235)	6.4 years	Estimated enterolactone, prediagnosis	postmenop. (all)	Q5 (median 502.0) vs. Q1 (median 146.0ug/d)	0.60 (0.40–0.89)	0.69 (0.43–1.10)		TNM, grade, ER/PR, mode of detection, diabetes, use of menopausal hormone therapy, study region, energy intake (strata: age)
Olsen 2012	Denmark, 1993–1997	424 (111/80)	10 years	Plasma enterolactone, prediagnosis	postmenop. (all)	>vs. ≤ 20.5 nmol/L(median)<br>per 20 nmol/L	0.47 (0.32–0.68)<br>0.82 (0.70–0.96)	0.56 (0.36–0.87)<br>0.88 (0.75–1.03)		grade, alcohol intake, menopausal hormone therapy

Table 2 Supplementary characteristics of studies

Study, Author, Publication Year (Ref)	Adjustment factors
LIBCSP Fink 2007[12]	age, energy intake
WEB McCann 2010[13]	age, education, race, stage at diagnosis, body mass index, and total energy intake

Guglielmini 2012[14]	menopausal status, tumor size, nodal status, adjuvant chemotherapy and adjuvant Tamoxifen
MARIE Seibold 2014[15]	TNM, grade, ER/PR, mode of detection, physical activity, time between blood draw and enterolact one measurement, menopausal hormone therapy, BMI, smoking, radiotherapy (strata: age, study region)
EPIC Cecilie 2015[16]	ER receptor status, cancer stage and grading of tumor. Further, strata are made for country and 5-year age group
Cecilie 2017[17]	smoking status at baseline (never, former, current), smoking intensity (packyears), schooling (short≤7 years, medium 8-10 years, long≥11 years), BMI at baseline (continuous, kg/m^2), physical activity measure at baseline (participate in sports, yes/no) and hormone use at baseline (never, former, current)

Newcastle-Ottawa Scale (NOS)

Author	Type of Study	Selection	Comparability	Outcome
Guglielmini 2012	retrospective cohort study	3	2	3
Cecilie 2015	Prospective multicenter cohort study	3	2	3
Cecilie 2017	Cancer and Health cohort	3	2	2

		Selection	Comparability	Exposure
Fink2007	case-control study	4	2	2
McCann2010	case-control study	3	2	2

Seibold 2014	case-control study	4	2	2

Search strategy

Embase

#1 mortality OR mortality:ti,ab,kw OR 'mortality'/exp OR survival OR survival:ti,ab,kw OR 'survival'/exp OR prognosis OR prognosis:ti,ab,kw OR 'prognosis'/exp OR (disease AND progression) OR 'disease progression':ti,ab,kw OR 'disease progression'/exp OR relapse OR relapse:ti,ab,kw OR 'relapse'/exp OR 'recurrent disease' OR 'recurrent disease':ti,ab,kw OR 'recurrent disease'/exp OR (treatment AND outcome) OR 'treatment outcome':ti,ab,kw OR 'treatment outcome'/exp OR death OR death:ti,ab,kw OR 'death'/exp

#2 enterolactone OR enterolactone:ti,ab,kw OR 'enterolactone'/exp OR lignan OR lignan:ti,ab,kw OR 'lignan'/exp

#3 ‘breast tumor’ OR ‘breast tumor’:ti,ab,kw OR ‘breast tumor’/exp

#4 #1 AND #2 AND #3

PubMed and Cochrane library

#1 (((((enterolactone[Title/Abstract]) OR (enterolactone)) OR (enterolactone[MeSH Terms])) OR (Lignans[MeSH Terms])) OR (Lignans)) OR (Lignans[Title/Abstract])

#2 (((((Breast Neoplasm[Title/Abstract]) OR (Breast Neoplasm)) OR (Breast Neoplasm[MeSH Terms])) OR (Mammary Cancers[MeSH Terms])) OR (Mammary Cancers)) OR (Mammary Cancers[Title/Abstract])

#3 ((((((((((((((((((((((mortality[MeSH Terms]) OR (mortality)) OR (mortality[Title/Abstract])) OR (survival[Title/Abstract])) OR (survival[MeSH Terms])) OR (survival)) OR (prognosis)) OR (prognosis[MeSH Terms])) OR (prognosis[Title/Abstract])) OR (disease progression[Title/Abstract])) OR (disease progression)) OR (disease progression[MeSH Terms])) OR (relapse[MeSH Terms])) OR (relapse)) OR (relapse[Title/Abstract])) OR (recurrence[Title/Abstract])) OR (recurrence)) OR (treatment outcome)) OR (treatment outcome[Title/Abstract])) OR (treatment outcome[MeSH Terms])) OR (death[MeSH Terms])) OR (death)) OR (death[Title/Abstract])

#4 #1 and #2 and #3

Web of Science

#1 Topic:(Breast Neoplasm) OR Title:(Breast Neoplasm) OR Topic:(Mammary Cancers) OR Title:(Mammary Cancers)

#2 Topic:(enterolactone) OR Title:(enterolactone) OR Topic:(Lignans) OR Title:(Lignans)

#3 Topic:(mortality) OR Title:(mortality) OR Topic:(survival) OR Title:(survival) OR Topic:(prognosis) OR Title:(prognosis) OR Topic:(disease progression) OR Title:(disease progression) OR Topic:(relapse) OR Title:(relapse) OR Topic:(recurrence) OR Title:(recurrence) OR Topic:(treatment outcome) OR Title:(treatment outcome) OR Topic:(death) OR Title:(death)

#4 #1 and #2 and #3

Figure 1 postmenopausal enterolactone in vivo and breast tumor recurrence

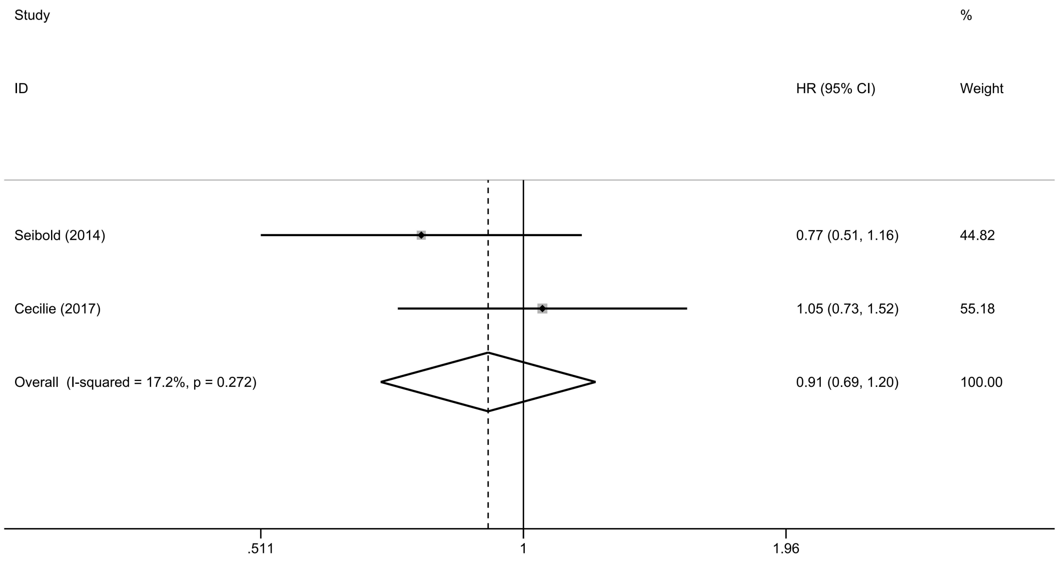

Postmenopausal lignans intake or enterolactone in vivo and risk of all− cause and breast cancer-specific mortality(upper quartile vs. minimum). (a) risk of all− cause mortality, (b) risk of breast cancer-specific mortality

(a)

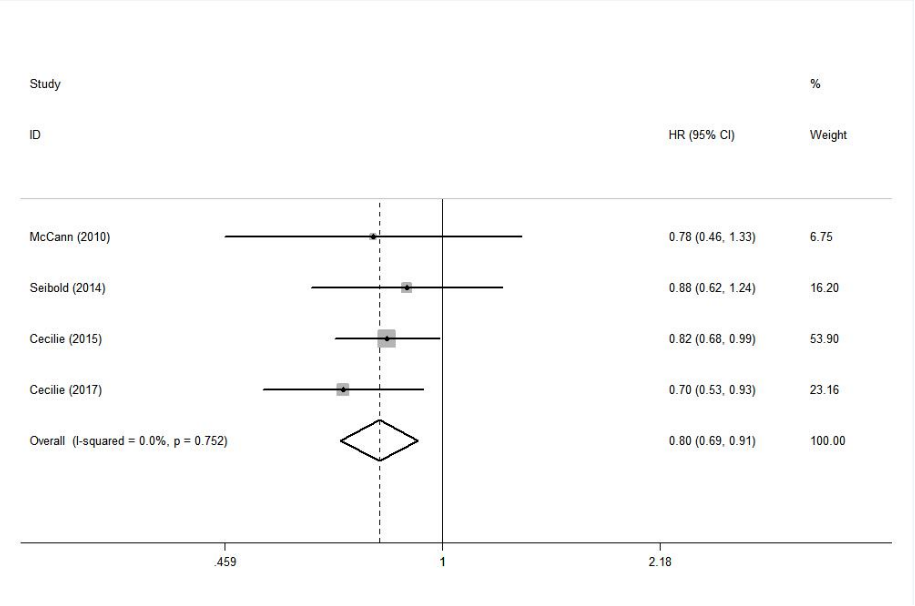

(b)

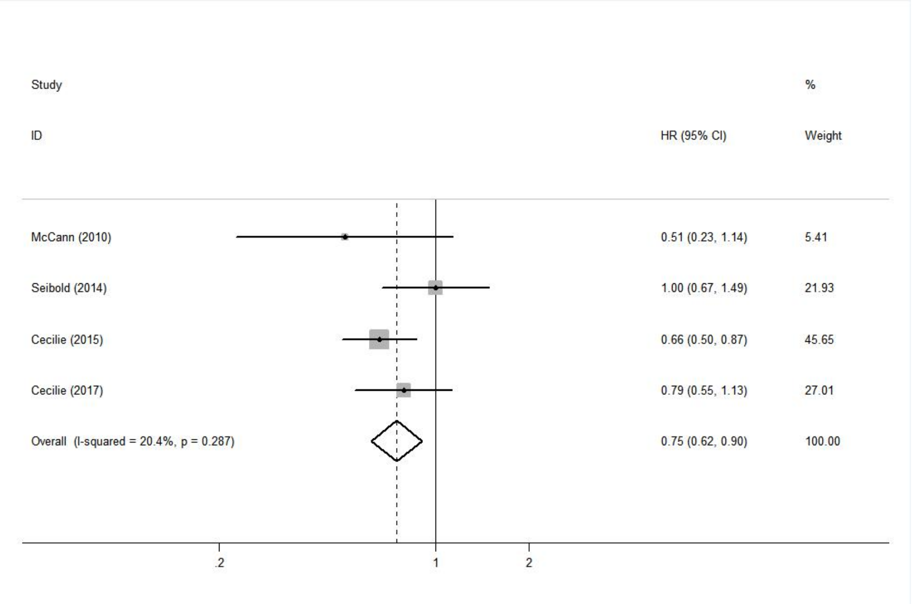

Postmenopausal lignans intake or enterolactone in vivo and risk of all− cause and breast cancer-specific mortality(lower quartile vs. minimum). (a) risk of all− cause mortality, (b) risk of breast cancer-specific mortality

(a)

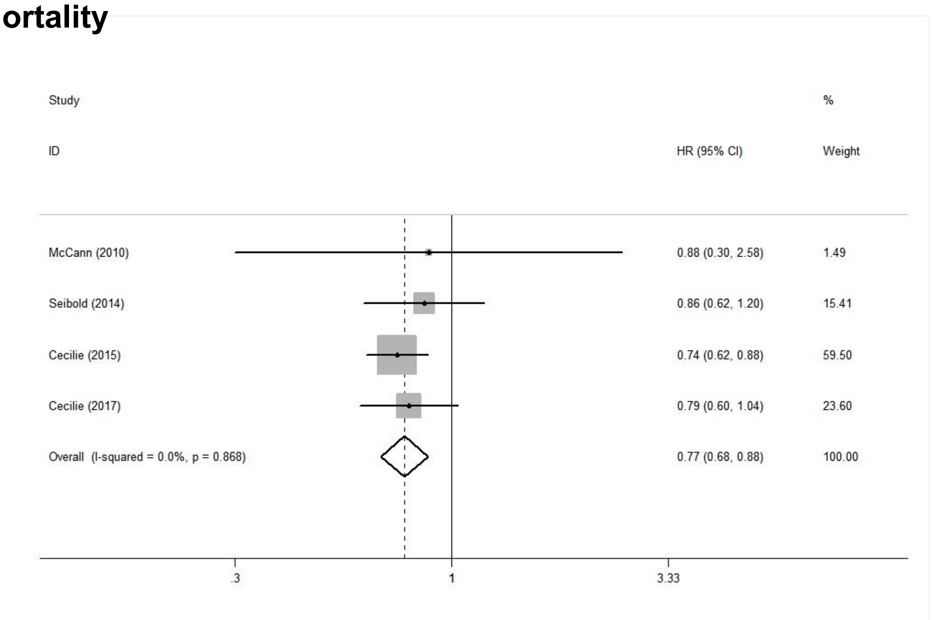

(b)

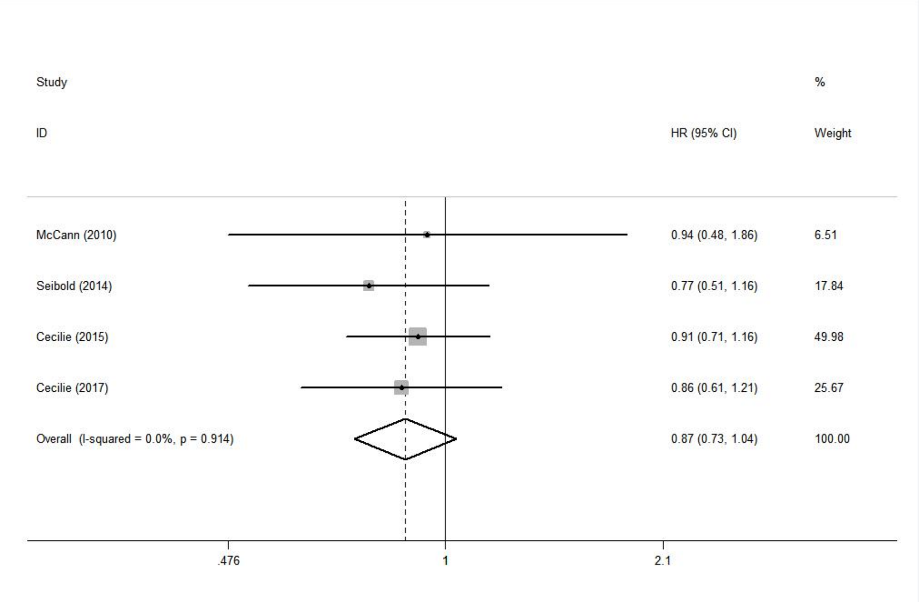

Premenopausal lignans intake or enterolactone in vivo and risk of all− cause and breast cancer-specific mortality(upper quartile vs. minimum). (a) risk of all− cause mortality, (b) risk of breast cancer-specific mortality

(a)

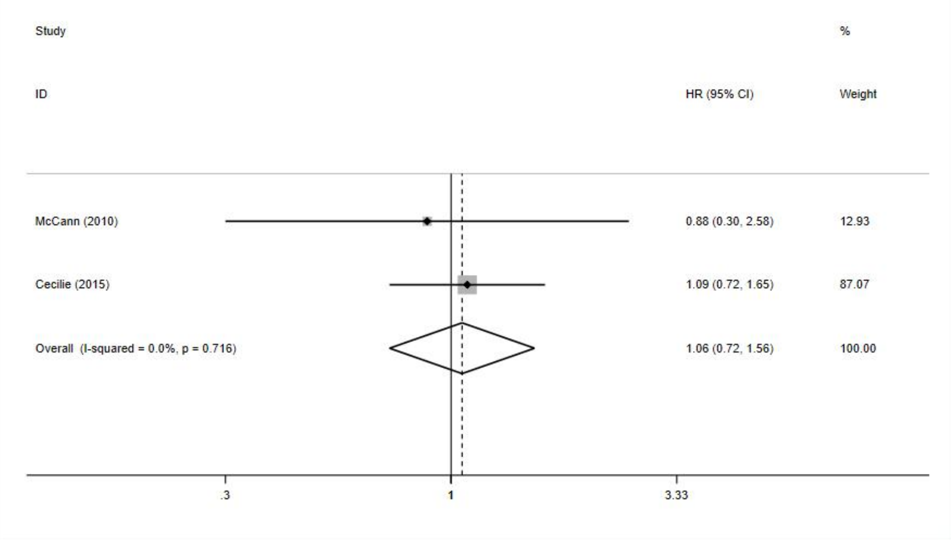

(b)

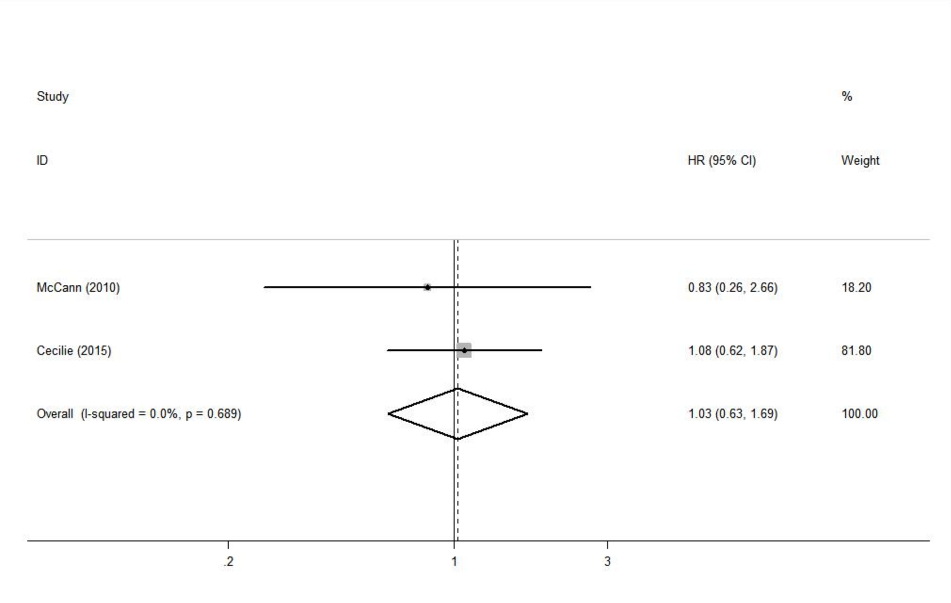

Premenopausal lignans intake or enterolactone in vivo and risk of all− cause and breast cancer-specific mortality(lower quartile vs. minimum). (a) risk of all− cause mortality, (b) risk of breast cancer-specific mortality

(a)

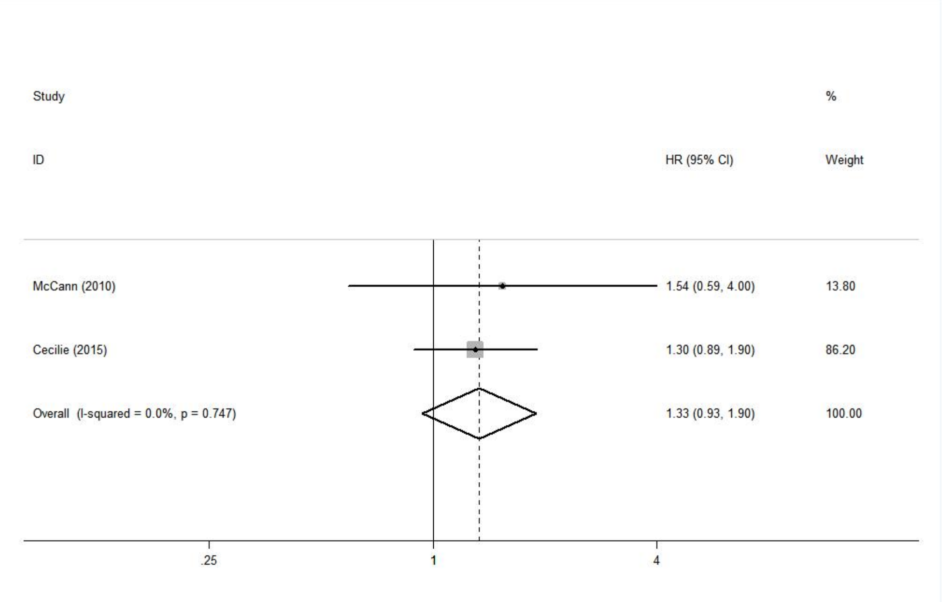

(b)

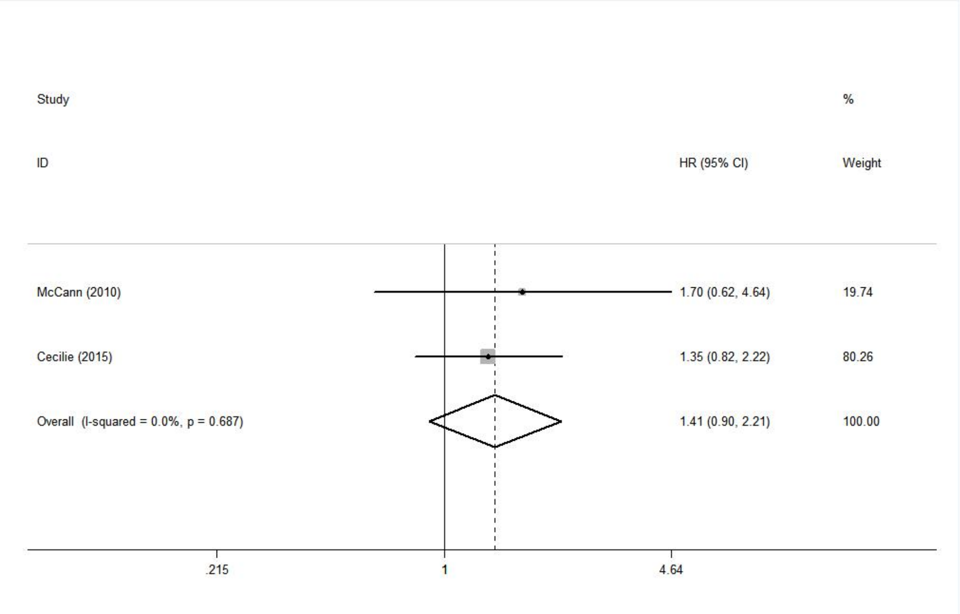

Supplement: Supplementary file 1 — Supplementary figures and tables. [file jcav12p2787s1.pdf]
